# Subunit Composition of Neurotransmitter Receptors in the Immature and in the Epileptic Brain

**DOI:** 10.1155/2014/301950

**Published:** 2014-09-11

**Authors:** Iván Sánchez Fernández, Tobias Loddenkemper

**Affiliations:** ^1^Division of Epilepsy and Clinical Neurophysiology, Department of Neurology, Boston Children's Hospital, Harvard Medical School, 300 Longwood Avenue Fegan 9, Boston, MA 02115, USA; ^2^Department of Child Neurology, Hospital Sant Joan de Déu, University of Barcelona, Passeig Sant Joan de Déu 2, Esplugues de Llobregat, 08950 Barcelona, Spain

## Abstract

Neuronal activity is critical for synaptogenesis and the development of neuronal networks. In the immature brain excitation predominates over inhibition facilitating the development of normal brain circuits, but also rendering it more susceptible to seizures. In this paper, we review the evolution of the subunit composition of neurotransmitter receptors during development, how it promotes excitation in the immature brain, and how this subunit composition of neurotransmission receptors may be also present in the epileptic brain. During normal brain development, excitatory glutamate receptors peak in function and gamma-aminobutiric acid (GABA) receptors are mainly excitatory rather than inhibitory. A growing body of evidence from animal models of epilepsy and status epilepticus has demonstrated that the brain exposed to repeated seizures presents a subunit composition of neurotransmitter receptors that mirrors that of the immature brain and promotes further seizures and epileptogenesis. Studies performed in samples from the epileptic human brain have also found a subunit composition pattern of neurotransmitter receptors similar to the one found in the immature brain. These findings provide a solid rationale for tailoring antiepileptic treatments to the specific subunit composition of neurotransmitter receptors and they provide potential targets for the development of antiepileptogenic treatments.

## 1. Introduction

Neuronal activity is critical for synaptogenesis and the development of neuronal networks. In the immature brain excitation predominates over inhibition facilitating the development of normal brain circuits. However, this tendency towards hyperexcitability also renders it more susceptible to seizures [1, 2]. Following prolonged seizures, the subunit composition of neurotransmitter receptors may be similar to that of the immature brain [1–6].

In this paper we will review the patterns of subunit composition of the main glutamate [α-amino-3-hydroxy-5-methyl-4-isoxazolepropionic acid (AMPA) and N-methyl-D-aspartate (NMDA)] and gamma-aminobutyric acid (GABA) receptors during development [7–13]. We will also review the subunit composition of neurotransmitter receptors that mirrors that of the immature brain, facilitating further seizures and the development of pathologic neuronal networks [14–21]. Finally, we will discuss the novel therapeutic targets that are being revealed by studying the subunit composition of the neurotransmitter receptors and potential therapeutic translation into clinical practice [3–6].

## 2. Subunit Composition of Glutamate and GABA_**A**_ Receptors in the Immature Brain (Table 1)

### 2.1. Data from the Immature Brain in Animal Models

**Figure 1:**
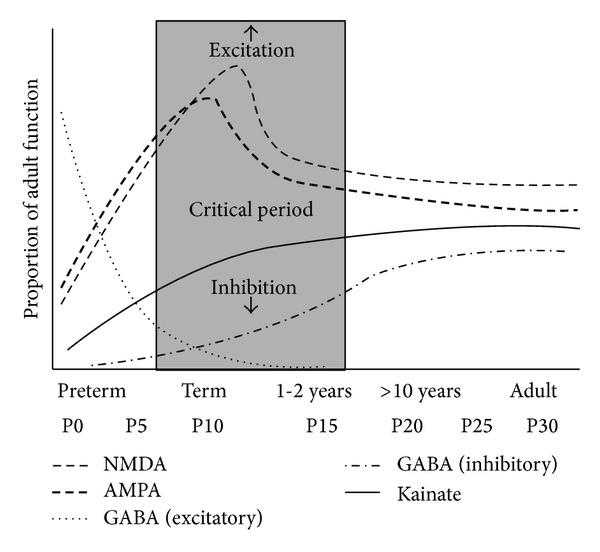
Evolution of neurotransmitter receptors expression over development. During the critical period (shaded rectangle), excitatory receptors are overexpressed, and inhibitory receptors are underexpressed compared to any other period of life. Approximate human ages are expressed in years (*x*-axis, upper row) and approximate rodent ages are expressed as postnatal days (*x*-axis, lower row). Legend: AMPA: alpha-amino-3-hydroxy-5-methyl-4-isoxazolepropionic acid. GABA: gamma-aminobutyric acid. NMDA: N-methyl-D-aspartate (adapted with permission from Rakhade and Jensen [1], by permission of Macmillan Publishers, Ltd., © 2009).

During normal brain maturation, the subunit composition of the AMPA, NMDA, and GABA_A_ receptors evolves over time and their function changes accordingly (Figure 1).

#### 2.1.1. AMPA Receptors

Reduced expression of the GluA2 subunit in AMPA receptors leads to an increased permeability to Ca^2+^ contributing to a lower threshold for seizures [2, 9, 24, 25]. AMPA receptors without the GluA2 subunit are typically expressed in the immature brain and their presence corresponds to an increased risk of excitotoxic cellular injury due to hypoxia-ischemia and of subsequent epileptogenesis both in rats and in humans [11, 12]. Knockout models of the GluA2 subunit lead to reduced seizure threshold and seizure-like behavior and, therefore, supports the hypothesis that AMPA receptors without the GluA2 subunit promote hyperexcitability [26].

#### 2.1.2. NMDA Receptors

In rat models of the immature brain GluN2B, GluN2C, GluN2D, and GluN3A subunits are overexpressed and this overexpression, which increases the ratio non-GluN2A/GluN2A, promotes hyperexcitability [1, 13, 25, 27].

#### 2.1.3. GABA_A_ Receptors and the Transporter of Chloride

**Figure 2:**
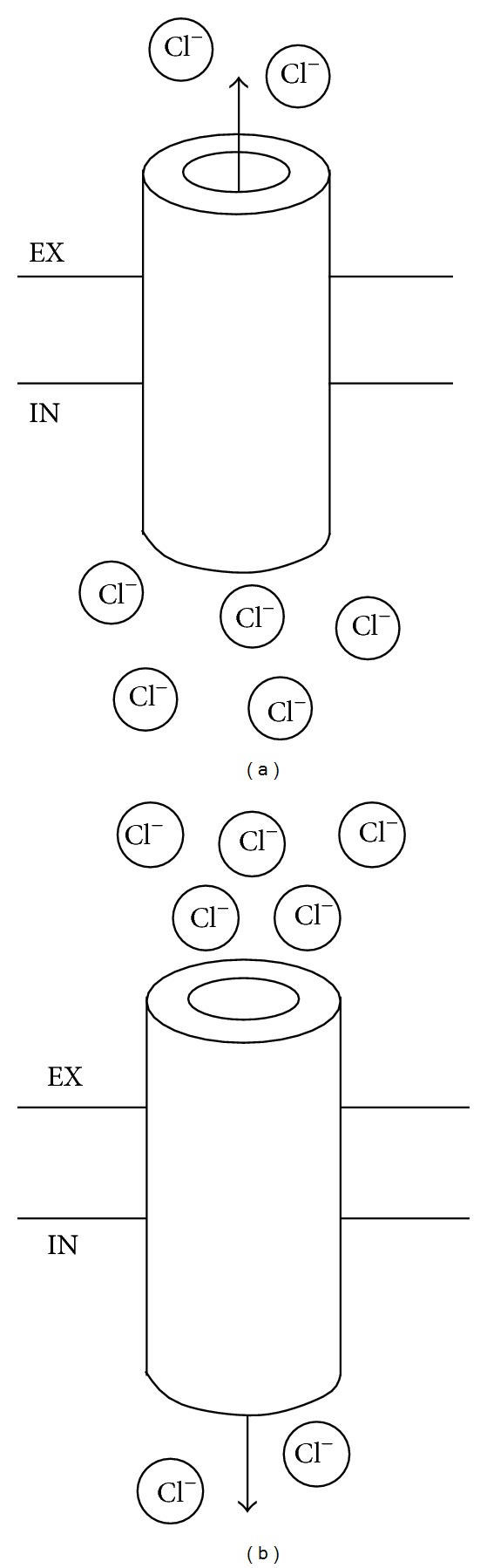
Effect of the opening of the GABA_A_ receptor associated channel. (a) In the immature neuron, the concentration of Cl^−^ is higher in the intracellular space and, therefore, the opening of the channel leads to an efflux of Cl^−^ and depolarization (excitation). (b) In contrast, in the mature neuron, the concentration of Cl^−^ is higher in the extracellular space and, therefore, the opening of the channel leads to an influx of Cl^−^ and hyperpolarization (inhibition). Legend: Cl^−^: chloride. IN: intracellular. EX: extracellular.

Contrary to what happens in mature neurons, in developing neurons the concentration of chloride (Cl^−^) is higher in the intracellular than in the extracellular space [28–30]. As a result, opening of GABA_A_ receptor associated Cl^−^ channel leads to different effects during development: an efflux of Cl^−^ and depolarization (excitation) in the immature neuron and an influx of Cl^−^ and hyperpolarization (inhibition) in the mature neuron (Figure 2) [28–30]. Cl^−^ concentration is mainly regulated by the action of the Cl^−^ transporters NKCC1 and KCC2. NKCC1 accumulates Cl^−^ in the cell and KCC2 transports Cl^−^ out of the cell. NKCC1 is the main Cl^−^ transporter in the immature neuron and its expression decreases over time while KCC2 is increasingly expressed during brain development [28, 29, 31–33]. KCC2 is the primary determinant of the changes in the gradients of Cl^−^ concentration during neuronal development and of the resulting maturation (from excitatory towards inhibitory) of GABAergic neurotransmission [32, 33]. While the major step during the maturation of GABA_A_ neurotransmission occurs during the shift in intraneuronal Cl^−^ concentration, changes in the subunit composition of GABA_A_ receptors may also play a role. Specifically, the α1 subunit of the GABA_A_ receptor is developmentally regulated and expressed at low levels in the immature brain [22].

### 2.2. Data from the Immature Human Brain 

#### 2.2.1. AMPA Receptors

When the ratio non-GluA2/GluA2 is elevated, the permeability to Ca^2+^ increases and this promotes hyperexcitability [34]. A pathologic study using immunoblotting and immunofluorescence compared the subunit composition of AMPA receptors of adults to that of fetuses, stillbirths, and newborns (gestational ages from 18 to 41 weeks). All of the study subjects died from nonneurological causes and presented no brain lesions or brain malformations. The non-GluA2/GluA2 ratio was elevated in fetuses, stillbirths, and newborns when compared to adults. In addition, this ratio peaked during early fetal development and preterm period in the white matter and during term and neonatal periods in the brain cortex, that is, during the periods of greater susceptibility to hypoxic-ischemic lesions in each component of the brain matter [12].

#### 2.2.2. GABA_A_ Receptors

In a series of patients who died without neurological diseases at different ages (from 36 weeks of gestational age to 81 years), the subunit composition of the GABA_A_ receptor was evaluated both by binding to benzodiazepines and by mRNA expression. A low level of expression of the α1 subunit was found in the immature (as compared to the mature) brain [7].

In summary, the immature brain has an increase of the following ratios: GluA1/GluA2 (AMPA receptors), GluN2B/GluN2A (NMDA receptors), NKCC1/KCC2 (GABA_A_ neurotransmission), and non-α1/α1 (GABA_A_ receptors) that promote hyperexcitability, synaptogenesis, and the development of normal neuronal networks in the developing brain [7, 9–13, 22]. A summary including the main features of the above-mentioned studies can be found in Table 1.

## 3. Subunit Composition of Glutamate and GABA Receptors in the Brain Submitted to Seizures (Table 2)

The subunit composition of neurotransmitter receptors in epilepsy and status epilepticus mirrors that of the immature brain [14–21]. Different animal models have shown that the brain exposed to recurrent seizures has an “immature” neurotransmission that may promote further seizures and epileptogenesis [14, 19]. These findings have been replicated in human brain tissue collected during epilepsy surgery [15–17, 20, 21].

### 3.1. Data from the Epileptic Brain in Animal Models

#### 3.1.1. AMPA Receptors

In a study of adult rats with pilocarpine-induced status epilepticus, the expression of surface GluA2 AMPA subunit was reduced [23]. In addition to changes in subunit composition, in a study of 4–7-week-old rats, AMPA receptors moved towards the cell surface and towards synapses during prolonged seizures further promoting an excitatory environment and self-sustaining seizures [35].

#### 3.1.2. NMDA Receptors

In an in vitro preparation of the hippocampus from 4-day-old rats, the induction of synchronized network discharges led to reduction in the expression of the subunits of the NMDA receptor, especially, a reduction of the GluN2A subunit [18]. In a 10-day-old rat model, tetanus toxin-induced seizures led to reduction in GluN1, GluN2A, and GluN2B subunits [19]. In a 9- to 13-day-old mouse model, flurothyl-induced seizures caused a reduction of the GluN2A subunit [19].

#### 3.1.3. GABA_A_ Receptors

In an adult rat model, the whole-cell patch-clamp recording technique showed that, following pilocarpine-induced status epilepticus, hippocampal neurons had GABA_A_ receptors with functional characteristics that promoted hyperexcitability [14]. This functional change was accompanied by a change in mRNA expression: mRNA for α1 subunits was decreased while mRNA for non-α1 subunits was increased [14]. This increase in the ratio of the mRNA for non-α1/α1 subunits was present soon after pilocarpine-induced status epilepticus and also several months later, when the rats had developed spontaneous temporal lobe seizures, suggesting that the increased ratio in the non-α1/α1 subunits is a common pathologic mechanism that occurs following seizures, regardless of etiology and duration [14]. In addition to changes in subunit composition, GABA_A_ receptors move away from synapses during prolonged seizures, increasing the excitatory/inhibitory balance and promoting self-sustaining SE [36].

### 3.2. Data from the Epileptic Human Brain 

#### 3.2.1. Origin of the Human Brain Samples: Epilepsy Surgery for Difficult-to-Control Seizures

Despite the large amount of data on subunit composition of glutamate and GABA receptors coming from the study of animal models of epilepsy, a validation in human brain tissue was necessary in order to apply these results into clinical practice [8, 14, 18, 19]. A major limitation to reproduce these studies in humans was the availability of fresh human brain tissue. However, epilepsy surgery has emerged as a useful therapeutic option for selected patients with refractory forms of epilepsy [37, 38] and it has opened a unique opportunity for the study of subunits in neurotransmitter receptors in the human brain [15–17, 20, 21, 37, 38]. The samples of fresh human brain tissue come from patients who underwent epilepsy surgery because of a heterogeneous group of epilepsy etiologies such as tuberous sclerosis complex [17, 20, 21] and other malformations of cortical development [15–17] and from patients with epilepsy of unknown etiology [15–17, 20]. Control tissue usually comes from autopsies of subjects who died from nonneurological causes [15, 17, 20, 21].

#### 3.2.2. Data from Patients with Tuberous Sclerosis Complex (TSC)

In patients with TSC, the subunit composition of neurotransmitter receptors mirrors that of the immature brain and may substantially contribute to increased network excitability in tubers and perituberal tissue [20, 21].

In an epilepsy surgery series, subunit composition of glutamate and GABA_A_ receptors was evaluated in patients with TSC, in cortex from epileptic patients without TSC, and in a control group of autopsy subjects without neurological diseases [20]. Brain tissue from TSC patients showed an elevation of the GluA1/GluA2 ratio (AMPA receptors) and an increase in GluN2B and GluN3A subunits (NMDA receptors) [20]. Cortex tissue from patients with epilepsy without TSC showed the same pattern of elevated GluA1/GluA2 ratio (AMPA receptors) and an increase in GluN2B and GluN3A subunits (NMDA receptors) [20].

In another study of human brain tissue, subunit composition was evaluated in patients with TSC, in cortex from epileptic patients without TSC, and in a control group of autopsy subjects without neurological diseases [21]. Patch-clamp studies of dysplastic neurons from patients with TSC showed a functional hyperexcitability of the GABA_A_ receptor [21]. Brain tissue from TSC patients and patients with focal cortical dysplasia type IIb showed reduction in the α1 subunit (GABA_A_ receptors) [21]. In addition, in patients with TSC and focal cortical dysplasia type IIb the expression of the Cl^−^ transporters mirrored that of the immature brain: increased NKCC1 and decreased KCC2 [21].

Taken together, these data show that in patients with TSC the neurons located in the tubers and in the perituberal tissue present a subunit receptor composition that mirrors that of the immature brain [20, 21].

#### 3.2.3. Data from Patients with Malformations of Cortical Development

Brain samples from patients with other malformations of cortical development also present an immature subunit composition of neurotransmitter receptors [15, 16].

In a study of mRNA expression in neurons from patients with malformations of cortical development, dysplastic neurons presented a reduction in mRNA expression for the GluA1 subunit and an elevation in the mRNA expression for the GluA4 subunit (AMPA receptor), an increase in the mRNA expression ratio for the GluN2B/GluN2A subunits (NMDA receptor), and a reduction in the mRNA expression for the α1 subunit (GABA_A_ receptor) [15]. In this same study, heterotopic, nondysplastic neurons showed a reduction in the mRNA expression for the GluA1 subunit (AMPA receptor) and a reduction in the mRNA expression for the α1 subunit (GABA_A_ receptor) [15].

In another study on the subunit composition of the NMDA receptors performed in patients with malformations of cortical development, tissue from focal cortical dysplasia showed increased GluN2B subunit and tissue from periventricular nodular heterotopia showed a reduction of GluN1, GluN2A, and GluN2B [16].

Taken together, these data point towards an immature subunit composition of neurotransmitter receptors in the abnormal, epilepsy-prone brain tissue of malformations of cortical development [15, 16].

#### 3.2.4. Data from Convulsive Status Epilepticus and from Electrical Status Epilepticus in Sleep

In vitro and in vivo animal models of refractory epilepsy and refractory status epilepticus have demonstrated that seizures, especially during early life, modify the expression patterns of glutamate and GABA_A_ receptors further enhancing hyperexcitability and leading to a brain tissue with a lower threshold for subsequent recurrent seizures and status epilepticus [1, 2, 8, 14, 18, 19, 39]. However, reproduction of these results in the human brain has been limited because of the limited availability of fresh brain tissue from patients with refractory epilepsy and refractory status epilepticus. A recent study has compared the subunit composition of glutamate and GABA receptors in brain samples from (i) patients with refractory convulsive status epilepticus, (ii) patients with refractory electrical status epilepticus during sleep (ESES), and (iii) patients with refractory epilepsy to the subunit composition neurotransmitter receptors of a control group of subjects who died of nonneurological causes [17]. Patients with refractory status epilepticus showed an increase in GluN2B subunit and GluN2B/GluN2A ratio (NMDA receptors) and an increase in the α2/α1 ratio (GABA_A_ receptors) [17]. Tissue from patients with refractory ESES showed an elevation of the GluA1 subunit and of the GluA1/GluA2 ratio (AMPA receptors) and increased GluN2B/GluN2A ratio (NMDA receptors) [17]. Brain samples from patients with refractory epilepsy showed an elevation of the GluA1/GluA2 ratio (AMPA receptors) and an elevation of the α2/α1 ratio (GABA_A_ receptor) [17]. The mRNA expression was also analyzed; patients with refractory convulsive status epilepticus had an elevation of the mRNA expression for the α2 subunit (GABA_A_ receptor) and patients with refractory epilepsy had an elevation of the mRNA for the α2 subunit and of the α2/α1 ratio (GABA_A_ receptor) [17]. All the changes found in subunit composition of glutamate and GABA_A_ receptors were consistent with “immature” “hyperexcitable” receptors, although because of low numbers only the above-mentioned cases reached statistically significant differences [17].

## 4. Limitations of the Studies on Subunit Composition of Neurotransmitter Receptors

### 4.1. Technical Limitations

#### 4.1.1. Immunofluorescence

Immunofluorescence identifies the individual subunit of interest in brain slices. This method provides information on the general distribution of the individual subunits in the brain. However, limitations of this technique include the unreliability of the calculation of the levels of expression of the individual subunits, and, therefore, it is impossible to compare the relative levels of expression of the different subunits from the same receptor.

#### 4.1.2. Subunit Levels of Expression

Immunofluorescence is often complemented with Western blotting [11, 12, 20, 21], a technique that allows calculating the levels of expression of neurotransmitter receptors and estimating the relative ratio of different subunits. However, subunit composition is studied in tissue samples and, therefore, its levels of expression not only reflect the levels of expression in the cellular membrane but also reflect its levels of expression in organelle's membranes. That is, a particular subunit could appear at normal levels in the studied tissue, but its levels of expression in the cellular membrane may be low (or high) because the subunit is kept in the membranes of the internal organelles [40]. Curiously, after a single episode of kainate-induced seizures in immature rats, there is a long-term shift of GluA1 receptor from the membrane to the intracellular compartment and a loss of total GluN2A in the rat hippocampus [40], changes that may increase hyperexcitability of the cellular membrane, but that would be undetectable when studying global tissue levels of expression.

#### 4.1.3. Timeline in the Evaluation of the Level of Expression of Subunit Composition of Neurotransmitter Receptors

Most studies present the results of changes in subunit composition of neurotransmitter receptors at a single point in time. Frequently, results refer to the acute-subacute period after seizure induction. Some series have a longer follow-up after seizure induction. Only a few report both results in the short and long term after seizure induction [14] and very few report changes in neurotransmitter receptors over time [41]. Despite the fact that the timeline for follow-up was different in various studies, we found a relatively consistent pattern of subunit composition changes in studies with different follow-up periods (Tables 1 and 2).

### 4.2. Limitations of the Studies in Animal Models

#### 4.2.1. Seizures Induced in the Immature Brain versus Seizures Induced in the Adult Brain

The age at which seizures are induced in animal models may influence the resulting changes in the subunit compositions. In fact, some animal models have demonstrated opposite changes in subunit expression in immature and adult animals [26]. However, most results show similar results in the immature and adult animal model (Tables 1 and 2).

#### 4.2.2. Experimentally Induced Seizures versus Naturally Occurring Seizures

Studies using animal models are based on the assumption that experimentally-induced seizures have a similar effect on the brain than naturally-occurring seizures. This assumption has been largely confirmed in most aspects of epilepsy research such as epileptogenesis [1]. However, to safely translate animal research findings into changes in clinical practice confirmation of results in human brain tissue is warranted.

#### 4.2.3. Applicability of Results from Animal Models to the Human Brain

The subunit composition of the “immature” and “hyperexcitable” AMPA receptor shows an elevation of the non-GluA2/GluA2 ratio both in animal models [9, 11] and in studies of the human brain [12, 17, 20]. The subunit composition of the “immature” and “hyperexcitable” GABA_A_ receptor shows an elevation of the non-α1/α1 ratio both in animal models [14] and in studies of the human brain [17, 21]. In contrast, the “immature” and “hyperexcitable” NMDA receptor has a decreased GluN2B subunit in rodent models [18, 19], while in humans the GluN2B subunit is elevated [15–17, 20]. The non-GluN2A/GluN2A ratio likely remains elevated in all these models.

### 4.3. Limitations of Human Brain Tissue Studies

#### 4.3.1. Heterogeneous Etiologies and Clinical Syndromes

Human brain tissue is collected from patients who undergo epilepsy surgery because of refractory epilepsy. Contrary to the animal models, the underlying etiology of refractory epilepsy is heterogeneous: malformations of cortical development, tubers from patients with TSC, or refractory epilepsy without an underlying structural etiology, among others [15–17, 20, 21]. Even within one broad category, such as malformation of cortical development, there is a wide variability of underlying pathologies with different patients presenting with different types of malformations. In addition, different cell populations from the same lesion are heterogeneous and may present with different patterns of subunit composition of neurotransmitter receptors [15, 16, 20].

#### 4.3.2. Limitations of the Control Subjects

Ideally, the best control tissue for patients that undergo epilepsy surgery should be a sample from the corresponding brain region in a nonepileptic and healthy subject of the same age. Obviously, that kind of control tissue is not available in human brain studies. Control tissue usually comes from autopsies of subjects who died from nonneurological causes [15, 17, 20, 21]. A rarer source of human brain tissue to be used as control tissue is the peritumoral tissue resected during tumor resection surgery [16].

#### 4.3.3. Limitations Associated with Heterogeneous Sampling Site

The localization of the brain tissue to be resected during surgery is decided pursuing the best interest of the patient. Therefore, this area is different among different patients and it is usually different from the area collected during autopsy in controls [16, 20, 21]. Potential regional variations in the expression of subunit composition of neurotransmitter receptors may compromise comparability of results.

Despite all the sources of heterogeneity most studies point in the same direction; subunit composition of neurotransmitter receptors in epileptic tissue mirrors that of the immature brain and promotes further hyperexcitability and epileptogenesis. Therefore, it seems reasonable to hypothesize that the “immature” subunit composition of neurotransmitter receptors is a common pathway that appears in response to seizures regardless of the etiology, clinical syndrome, or sampled brain area.

#### 4.3.4. Number of Study Subjects

The previous limitations can be potentially overcome if there were enough study subjects to compare the different subgroups (e.g., only patients with status epilepticus secondary to focal cortical dysplasia in the temporal lobe). However, collection of a large number of human brain samples is limited by ethical and practical reasons. Human series on this topic most frequently consist of less than 10 patients in each group [15–17, 20, 21]. Even a large autopsy study of perinatal patients who died of nonneurological causes in a large reference hospital collected “only” 43 patients and 3 adult controls [12]. Taken together, these data highlight that even for large reference centers it is not feasible to collect enough patients to further develop this line of research.

## 5. Outlook: The Need for Multicenter Collaboration

Collecting a very large number of brain samples with homogeneous pathology and comparable characteristics is not a feasible goal for any individual hospital or group of hospitals. Only a multicenter collaboration with the development of national or international centralized tissue banks may sum up the numbers necessary to achieve this goal. The University of Maryland Tissue Bank is a human brain samples repository that is currently collecting brain tissue samples from different pathologies and may be useful for the study of subunit composition of neurotransmitter receptors in epilepsy.

## 6. Clinical Implications

In the developing brain there is a predominance of excitation over inhibition in order to meet the needs of the rapid development of synapses and neuronal networks (Figure 1) [1, 2, 42]. This tendency towards hyperexcitability is reflected in the subunit composition of the normal immature brain both in animal models [9–11, 13] and in samples from the perinatal human brain [12]. As the brain matures and the need for the development of synapses and neuronal networks decreases, the tendency towards hyperexcitability decreases and the subunit composition changes and acquires its adult features (Figure 1) [1, 2]. However, when exposed to repeated seizure activity, the brain tissue goes back to a subunit composition and distribution of neurotransmitter receptors that mirrors that of the immature brain and the enhanced synaptogenesis promotes the development of pathologic neuronal networks (epileptogenesis) [1, 2, 43, 44].

Differences in neurotransmission should be taken into account when treating epileptic patients. Antiepileptic medications act differently in the mature and immature brain tissue. As an example, GABAergic anticonvulsants such as phenobarbital and benzodiazepines can have a dual action in the immature brain: both excitatory and inhibitory [4]. The excitatory response is secondary to the high concentration of intracellular Cl^−^ in the immature neuron mainly due to the action of the NKCC1 transporter that accumulates Cl^−^ intracellularly [28–30]. Blocking the NKCC1 transporter with bumetanide enabled the antiepileptic action of phenobarbital in vitro [3]. Similarly, in an in vitro model of seizures, phenobarbital was able to control seizures when applied from the start but aggravated seizure activity when applied after repeated seizures (that had probably changed the chloride concentrations in the affected neurons) [6]. Application of bumetanide in this model prevented the paradoxical actions of phenobarbital [6] and trials are ongoing in an attempt to translate findings into neonates. Taken together, these results suggest that the knowledge on the subunit composition of neurotransmitter receptors may allow the development of a targeted, pathophysiology-based use of antiepileptic medications.

Another example of the application of this knowledge into clinical practice is status epilepticus; status epilepticus responds to most agents early in the course of the disease but tends to be quite refractory once it is established [36, 45–48]. During status epilepticus benzodiazepine sensitive GABA_A_ receptors are believed to move from the synaptic membrane to the cytoplasm where they are functionally inactive while NMDA and AMPA receptors move from subsynaptic sites to the synaptic membrane [36, 45–48]. In addition, refractory status epilepticus promotes the development of a subunit composition of neurotransmitter receptors pattern that mirrors that of the immature brain [17]. These underlying molecular mechanisms are consistent with findings in experimental models that GABAergic antiepileptic drugs lose their efficacy early in the course of status epilepticus [49] but NMDA receptor blockers suppress seizures even late in the evolution of status epilepticus [5]. As a consequence, NMDA receptor blockers were proposed as the optimal therapy for refractory status epilepticus, with some success [5]. A detailed knowledge of the underlying pathophysiology may allow development of more individualized treatment strategies.

The study of the subunit composition of the neurotransmitter receptors in the epileptic brain is a developing field of research that promises to provide new therapeutic targets for seizures and epileptogenesis.

## 7. Conclusion

The subunit composition of neurotransmitter receptors promotes excitation over inhibition in the developing brain. Because neuronal activity is critical for synaptogenesis and the development of normal neuronal networks, this tendency towards excitation facilitates the development of normal brain circuits. Under conditions of repeated epileptiform activity, the subunit composition of AMPA, NMDA, and GABA receptors is similar to that of the developing brain, promoting further seizures and the development of pathological neuronal networks (epileptogenesis). This developing field of research promises to unravel new therapeutic targets for the treatment of seizures and epileptogenesis.

## Figures and Tables

**Table 1:** Subunit composition of glutamate and GABA receptors in the immature brain.

Author and year	Study design	Substudy features (if applicable)	AMPA	NMDA	GABA
Animal models of the immature brain without seizures					
Kumar et al., 2002 [9]	Neocortical pyramidal neurons in excitatory layer 5, immature nonepileptic rats (compared to mature rats)		GluA2 ↓ GluA1/GluA2 ↑ GluA4/GluA2 ↑		

Talos et al., 2006 [11]	Immature nonepileptic rats (compared to mature rats)	White matter	GluA1 ↓ GluA2 ↓ GluA3 ↑ GluA4 ↑ nonGluA2/GluA2 ↑		
		Gray matter	GluA1 ↑ GluA2 ↓ GluA3 ↓ GluA4 ↓ nonGluA2/GluA2 ↑ GluA1/GluA2 ↑		

Monyer et al., 1994 [10]	Immature nonepileptic rats (compared to mature rats). Various areas in the brain			GluN2A ↓ GluN2B ↓ GluN2C ↓ GluN2D ↑	

Wong et al., 2002 [13]	Immature nonepileptic rats (compared to mature rats). Various areas in the brain			GluN3A ↑ GluN3A/GluN1 ↑	

Dunning et al., 1999 [22]	Functional studies of neurons from somatosensory cortex of neonatal mice				*α*1 ↓

Data from the human immature brain without seizures					
Talos et al., 2006 [12]	Autopsy samples from newborns of different gestational ages (compared to adult standard). Parietooccipital lobe tissue. Death due to nonneurological disorders	White matter	GluA2 ↓ GluA1/GluA2 ↑ GluA3 ↑ GluA3/GluA2 ↑ GluA4 ↑ GluA4/GluA3 ↑		
		Gray matter	GluA1 ↑ GluA2 ↓ GluA1/GluA2 ↑ GluA3 ↓ GluA4 ↓		

Brooks-Kayal and Pritchett, 1993 [7]	Autopsy samples from patients without neurological disease of different ages (36 gestational weeks to 81 years). Frontal cortex and cerebellum				*α*1 ↓ *α*1 ↓∗

*Studies on mRNA.

**Table 2:** Subunit composition of glutamate and GABA receptors in the brain submitted to seizures.

Author and year	Study design	Substudy features (if applicable)	AMPA	NMDA	GABA
Animal models of seizures and status epilepticus					
Brooks-Kayal et al., 1998 [14]	Rats with pilocarpine-induced status epilepticus and subsequent development of spontaneous temporal lobe seizures (compared to control rats). Hippocampus	24 hours after SE			α1 ↓∗ α3 ↑∗ α4 ↑∗ α1/non-α1 ↓∗ *β*1 ↓∗ *β*3 ↑∗ *δ* ↑∗ *ε* ↑∗
		1–4 months after SE and with spontaneous temporal lobe seizures			α1 ↓∗ α4 ↑∗ α1/non-α1 ↓∗ *δ* ↑∗ *ε* ↑∗ *β*1 ↓∗ *β*3 ↑∗

Swann et al., 2007 [19]	Rats with tetanus toxin or flurothyl-induced seizures (compared to control rats)	Tetanus toxin-induced seizures at p10 in hippocampus		GluN1 ↓ GluN2A ↓ GluN2B ↓	
		Flurothyl-induced seizures in hippocampus		GluN2A ↓	
		Flurothyl-induced seizures in neocortex		GluN2A ↓	

Rajasekaran et al., 2012 [23]	Rats with pilocarpine-induced status epilepticus (compared to control rats)		GluA2 surface expression ↓		

Data from epilepsy surgery performed for refractory epilepsy					
Crino et al., 2001 [15]	Individual neurons from dysplastic tissue from epilepsy surgery (compared to nondysplastic tissue from epileptic patients and to autopsy specimens from patients who died from nonneurological causes). Temporal neocortex and dorsolateral frontal neocortex	Dysplastic neurons	GluA1 ↓∗ GluA4 ↑∗	GluN2A ↓∗ GluN2B ↑∗ GluN2C ↑∗	α1 ↓∗ α2 ↓∗ *β*1 ↓∗ *β*2 ↓∗
		Heterotopic neurons	GluA1 ↓∗		α1 ↓∗ α2 ↓∗ *β*2 ↓∗

Talos et al., 2008 [20]	Patients with tuberous sclerosis complex and epilepsy who underwent epilepsy surgery (compared to patients with epilepsy without tuberous sclerosis and to autopsy cases without neurological diseases)	Tissue from tubers	GluA1 ↑ GluA4 ↑ GluA2 ↓ GluA3 ↓	GluN2B ↑ GluN3A ↑	
		Cortex from epileptic patients without tuberous sclerosis	GluA1 ↑ GluA2 ↓	GluN2B ↑ GluN3A ↑	

Talos et al., 2012 [21]	Patients with tuberous sclerosis complex and epilepsy who underwent epilepsy surgery or whose tissue was collected at autopsy and patients with focal cortical dysplasia and epilepsy that underwent epilepsy surgery to resect the epileptogenic tissue (compared to autopsy cases without neurological diseases)	Tubers			α1 ↓ α4/α1 ↑
		Focal cortical dysplasia IIa			α4 ↓ α4/α1 ↓
		Focal cortical dysplasia IIb			α1 ↓ α4/α1 ↑

Finardi et al., 2006 [16]	Patients with malformations of cortical development undergoing epilepsy surgery because of refractory epilepsy (compared to patients with focal epilepsy without underlying malformation and to nonepileptic patients, brain tissue resected next to a tumor)	Focal cortical dysplasia		GluN2B ↑	
		Periventricular nodular heterotopia		GluN1 ↓ GluN2A ↓ GluN2B ↓	

Data from epilepsy surgery performed for refractory status epilepticus					
Loddenkemper et al., 2014 [17]	Patients with SE and ESES (compared to epilepsy surgery patients without status epilepticus, EPI, and to autopsy cases)	SE		GluN2B ↑ GluN2B/GluN2A ↑	α2/α1 ↑ α2 ↑∗
		ESES	GluA1 ↑ GluA1/GluA2 ↑	GluN2B/GluNA ↑	
		EPI	GluA1/Glu2 ↑		α2/α1 ↑ α2 ↑∗ α2/α1 ↑∗

*Studies on mRNA.
